# The Efficacy of Low-Dose Risperidone Treatment for Post-Surgical Delirium in Elderly Orthopedic Patients

**DOI:** 10.3390/medicina59061052

**Published:** 2023-05-30

**Authors:** Lotan Raphael, Epstein Edna, Kaykov Irina, Hershkovich Oded

**Affiliations:** Department of Orthopedic Surgery, Wolfson Medical Center, Sackler School of Medicine, Tel Aviv 5822012, Israel

**Keywords:** delirium, risperidone, post-surgical, elderly, protocol

## Abstract

***Background***: Delirium is an acute and typically reversible failure of essential cognitive and attentional functions and is a growing public health concern, with an incidence of 20–50% in patients older than 65 after major surgery and 61% in patients undergoing hip fracture surgery. Numerous treatment strategies have been examined with no conclusive results. The purpose of this study is to assess the efficacy of a three-day low-dose risperidone treatment protocol, 0.5 mg BID, in treating delirium in elderly hospitalized orthopedic surgery department patients. ***Methods***: This study is a prospective non-randomized study involving the senior patient population, older than 65, in an Orthopedic Surgery Department in 2019 and 2020. Delirium was diagnosed by a confusion assessment method (CAM) questionnaire. A three-day 0.5 mg risperidone BID treatment protocol was initiated following diagnosis. Patient data collected included age, gender, chronic diseases, type of surgery and anesthesia and delirium characteristics. ***Results***: The delirium study group included 47 patients with an average age of 84.4 years (±8.6), of whom 53.2% were females. Delirium incidence was 3.7% in all patients older than 65 (1759 patients) and 9.3% in the proximal femoral fracture group. We did not correlate electrolyte imbalance, anemia, polypharmacy and chronic diseases to delirium onset characteristics. Following the three-day low-dose risperidone treatment protocol, 0.5 mg BID, 14.9% of the patients showed CAM score normalization after one day of treatment, and 93.6% within two days. ***Conclusions***: We found our rigid three-day low-dose risperidone treatment protocol, 0.5 mg BID, efficacious in fast delirium resolution, without side effects.

## 1. Introduction

Delirium is an acute and typically reversible failure of essential cognitive and attentional functions. Delirium is usually associated with an altered fluctuating level of consciousness. It can manifest as agitation (hyperactive type), lethargy (hypoactive type), or alternating between these (mixed type). With the increase in life expectancy and the ageing population, many frail elderly patients require surgery, such as osteoporotic proximal femur fractures. Postoperative delirium is a growing public health concern, with an incidence of 20–50% in patients older than 65 after major surgery and 61% in patients undergoing hip fracture surgery [1,2]. Postoperative delirium can become the most deleterious element of the perioperative experience, both for the patient and the family, and is associated with increased mortality [1], cognitive and functional decline [2], increased hospital length of stay, and healthcare costs. The pathophysiology of delirium remains obscure, and the diagnosis is clinical.

Delirium is currently diagnosed according to the Diagnostic and Statistical Manual of Mental Disorders (DSM-V) or the 10th revision of the International Statistical Classification of Diseases and Related Health Problems (ICD-10). Faster and simpler delirium screening tools were described, including the confusion assessment method (CAM) [3,4,5], the delirium observation screening scale, delirium symptom interview, and the NEECHAM confusion scale [6], with low sensitivity (about 30%) compared with an expert delirium assessment (that is, by a psychiatrist, geriatrician, or neurologist) [7,8]. However, diagnostic disagreement may be typical among experts and disciplines. Whatever tool is used to diagnose delirium, a reassessment should be repeated every 6 h for three to five days following surgery.

Postoperative delirium risk factors include age greater than 65 years, preoperative cognitive impairment, visual or hearing impairment, presence of infection, inadequate or exaggerated pain control, impaired left ventricular function, electrolyte disorders, depression, alcoholism, smoking, high perioperative transfusion requirements, intraoperative pressure fluctuation [9], sleep deprivation, urinary retention or constipation, renal insufficiency, poor nutrition, dehydration, immobilization [10] or poor mobility, use of anticholinergic medications, sedative-hypnotics, and meperidine. Polypharmacy (five drugs or more) is another risk factor for delirium.

Although postoperative delirium is quite a common diagnosis, treatment is lacking. Delirium management starts with prevention, is multidisciplinary, and deals with 10 aspects: cerebral oxygenation, fluid, and electrolyte homeostasis, pain control, reduction of psychoactive drugs, bowel and urinary function optimization, nutritional support, early out-of-bed practice, prevention of postoperative complications, keeping adequate environmental stimuli, and treatment of delirium symptoms. Delirium management can be non-pharmacological or pharmacological [3,11,12,13].

Non-pharmacological delirium management focuses on mediating a calm and quiet patient environment, clock and calendar patient orientation, including familiar relatives, reducing treating staff rotation, “education about early mobilization”, reducing noise and lighting during night hours and opening windows and curtains during daylight hours, as well as improving patient communication by using hearing and visual aids. Mindful breathing also showed a 15–50% decrease in delirium incidence [14].

Intraoperative measures include light instead of heavy sedation and bispectral index (BIS) anesthesia guidance [15]. The role of regional versus general anesthesia in delirium remains unknown [16,17,18]. Postoperative pain control uses non-opiate alternatives instead of opiate-only regimens or combining regional anesthesia [13,19]. Gabapentin has been used in postoperative pain control but was found to be highly deliriogenic [20]. Postoperative measures include reviewing pain control and medications, physical examination with urinary catheter insertion if needed, and searching for an infection (pneumonia, sepsis, line sepsis, surgical site infection), and metabolic derangement.

Use of preventive antipsychotic medications and cholinesterase inhibitors is inconclusive. Some RCT studies showed that antipsychotic drugs, such as haloperidol, are efficacious in treating existing delirium, with side effects such as extrapyramidal manifestations and QT-interval elongation on ECG [21,22]. Their use is limited in patients suffering from hepatic insufficiency neuroleptic malignant syndrome. Another treatment option is atypical antipsychotic medications, such as risperidone or olanzapine, with the same side effects [23]. Risperidone stands out due to its notable affinity with serotonin and dopamine receptors. By exerting antagonistic effects on serotonin receptors, it can alleviate dopamine antagonism associated with extrapyramidal symptoms. As a result, risperidone exhibits superior advantages in terms of side effects compared to traditional antipsychotics, making it a preferred first-line treatment option for delirium [24,25]. Regardless of the treatment, the lowest effective dose for the shortest possible duration should be used to treat severely agitated or distressed patients that threaten themselves or their surroundings.

While numerous treatment strategies were examined with no conclusive results, this study aims to assess the efficacy of a three-day low-dose risperidone treatment protocol, 0.5 mg BID, in treating delirium in elderly hospitalized orthopedic surgery department patients.

## 2. Methods

This study is a prospective observational non-randomized study involving the senior patient population in an Orthopedic Surgery Department in 2019 and 2020. The study included patients older than 65 who suffered from delirium. A confusion assessment method (CAM) questionnaire diagnosed the patient’s delirium as evident from the presence or absence of the following four features: 1. mental status alteration from baseline (acute onset or fluctuating), 2. inattention, 3. disorganized thinking, and 4. altered level of consciousness. Delirium was identified only if there was evidence of the first essential features (1 + 2) and either of the following features (3 or 4, or both). Delirium was treated by cessation of opiates and psychoactive drugs and received a three-day BID treatment of 0.5 mg risperidone. This treatment protocol was chosen due to its simplicity, low dosage and short treatment period. Exclusion criteria included patients younger than 65 or patients who did not meet the CAM criteria for delirium or did not complete the three-day treatment protocol.

Patient data collected included age, gender, chronic diseases, type of surgery, type of anesthesia, type of anesthetic agents used during surgery, the timing of the delirium onset, delirium duration, and CAM scores at the protocol completion.

Statistical analysis included the Shapiro–Wilk test of normal distribution and students *t*-test for numerical parameters, and the Chi-square test for categorical parameters. In addition, Pearson and Spearman’s correlation coefficients measured the influence of gender, drugs, type of surgery, and anesthesia on delirium onset and duration. To counteract the multiple comparisons issue and the increase in probability of observing a rare event under multiple hypotheses, a Bonferroni correction was used as required.

## 3. Results

A total of 47 patients 65 years old or older out of the 1759 patients admitted to the Orthopedic Surgery Department between March 2019 and December 2020 suffered from perioperative delirium, in other words, 3.7% of the patients. The number of patients suffering proximal femoral fractures during the same period was 698; thus, there was 9.3% of delirium in this group of patients. The average age was 84.4 years (±8.6), and 53.2% were females. The average body mass index (BMI) was 25.4 (±6.4) (Table 1).

Some 80% of the patients in this study had proximal femoral fractures, which could be divided into subcapital (37.1%) and intertrochanteric fractures (44.7%). Three patients suffered pelvic fractures, pubic rami fractures that did not require surgery and were treated with physiotherapy, and deep vein thrombosis prevention and pain control. Six patients developed delirium following other surgeries; one case of open reduction internal fixation (ORIF) of a humeral fracture, one case of ORIF of a distal femoral fracture, one case of lumbar decompression laminectomy, one case of ORIF of an olecranon fracture and two cases of late femoral surgical site infection (SSI) debridement. General anesthesia accounted for 61.4% of surgeries, and regional anesthesia with sedation accounted for 38.6%.

**Figure 1:**
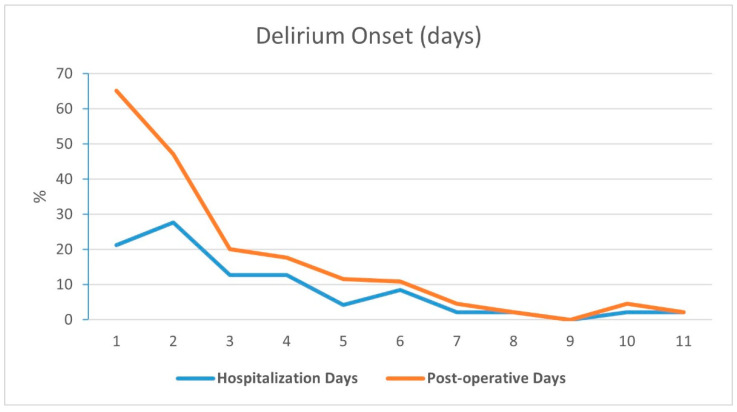
Delirium onset by days from hospitalization and postoperative time.

Delirium assessment was carried out once in a nursing shift, i.e., three times a day. The average CAM score at delirium onset was 3 ± 1, and patients tended to develop perioperative delirium during the evening (18:00–24:00 p.m.), but it did not reach statistical significance. A total of 63.8% of delirium onset occurred during the first three days of hospitalization and 76.6% during the first four days (Figure 1). In addition, 75% of the delirium occurred within the first two days following surgery and 81.8% during the first three postoperative days. In our study, females tended to develop delirium earlier during hospitalization (2.45 days ± 1.68) compared to males (5.16 days ± 7.41) (*p* = 0.05).

Examining factors influencing delirium onset, duration and severity, gender but not age, correlates with an earlier development of perioperative delirium (*p* = 0.046) and a tendency to prolonged delirium (*p* = 0.058). Unfortunately, the study sample size did not allow statistical significance for the correlation between gender and the duration of delirium.

**Figure 2:**
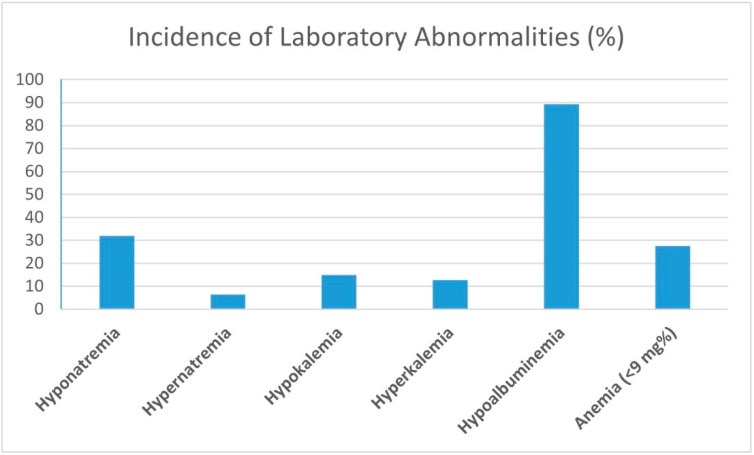
Incidence of laboratory abnormalities (%).

Opiates, benzodiazepine, anxiolytics and antidepressant treatments were not found to influence the rate of development of perioperative delirium in our study group or the effect of the treatment protocol. A history of malignancy, CVA, depression, and dementia did not change the course of the perioperative delirium either. Smoking did not correlate to the study group’s delirium characteristics. Common chronic diseases, such as diabetes, ischemic heart disease, and hypercholesterolemia, did not correlate to delirium onset or duration. Chronic renal failure, even when requiring dialysis, did not alter the delirium manifestation (Figure 2).

**Figure 3:**
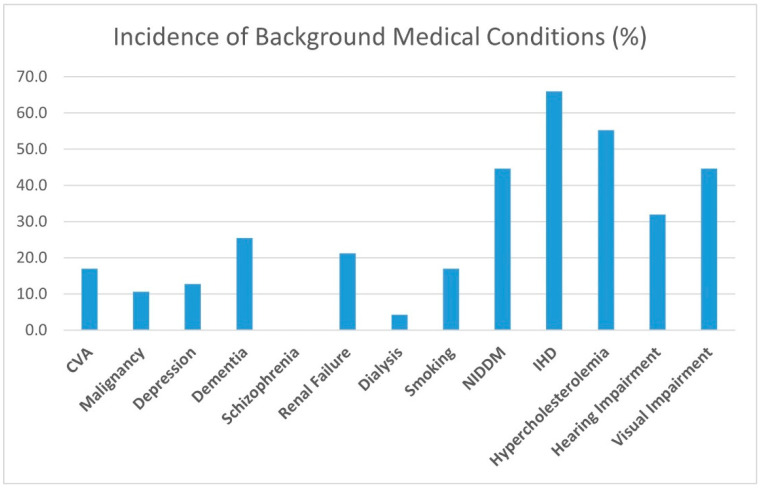
Incidence of background medical conditions.

Sensual impairments, visual and auditory, did not correlate to delirium duration, severity or onset. Blood electrolyte disturbances, such as hypo- and hyper-natremia and kalemia (Figure 3), did not influence perioperative delirium. Constipation and urinary retention did not correlate to the study group’s delirium onset, severity, and duration measured by Pearson and Spearman’s correlation coefficients.

Polypharmacy (more than five drugs) in the study group showed a low correlation to the duration of the perioperative delirium (r = 0.16), while higher blood albumin levels (r = −0.17) and younger age (*p* = 0.03) shortened the delirium period.

When we examined the effect of the type of anesthesia on postoperative delirium, we found that patients tended to develop delirium earlier following regional anesthesia with sedation (1.75 ± 2.41 days) as compared to general anesthesia (2.69 days ± 2.41 days) (*p* = 0.02). However, delirium following general anesthesia tended to last longer (2.15 ± 0.77 days) compared to regional anesthesia (1.24 ± 2.17 days) (*p* = 0.05).

Duration of surgery in the study group did not correlate to the onset, severity and duration of postoperative delirium. We found a trend towards earlier delirium onset in the arthroplasty group than the fracture fixation, but it did not reach statistical significance due to the sample size. There was no difference between hip arthroplasty and femoral fracture fixation groups regarding the duration of postoperative delirium. In this study, surgeries other than proximal femoral fractures developed more extended delirium periods than those of femoral fractures (2.71 ± 1.25 days vs. 1.87 ± 0.43 days, respectively) (*p* = 0.01).

The time between surgery and the first physiotherapy treatment correlated to delirium development (r = 0.34, *p* = 0.01). A possible bias that can explain this correlation is the patient’s post-surgical status; patients with lower vital signs or postoperative hemoglobin levels are not treated by physiotherapists until considered stable.

**Figure 4:**
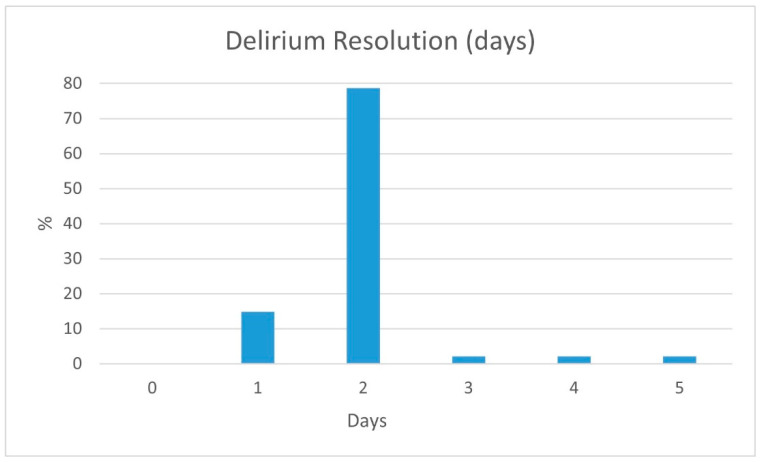
Time to delirium resolution (days).

Following the three-day low-dose risperidone treatment protocol, 0.5 mg BID, 14.9% of the patients showed a CAM score normalization after one day of treatment, and 93.6% within two days (Figure 4). However, 6.3% of the patients needed up to five days to normalize their CAM scores. None of the study’s patients suffered delirium for more than a five-day total following the three-day low-dose risperidone treatment protocol.

None of the delirium study group’s patients ceased the three-day low-dose respiridone protocol due to delirium escalation requiring a higher respiridone dose or a different medication. The only excluded patients not described in this study were a few requiring hospitalizations in another department due to acute medical conditions such as post-resuscitation, upper gastrointestinal bleeding, or rapid atrial fibrillation.

Study group patients were monitored for low dose risperidone treatment protocol adverse effects, such as nausea, vomiting, diarrhea or constipation, heartburn, dry mouth, increased salivation, stomach pain, anxiety, agitation, restlessness, vision impairment, muscle or joint pain, dry or discolored skin, difficulty urinating, confusion, tachycardia or arrhythmias, seizures, rash and difficulty breathing or swallowing. No adverse effects were registered.

## 4. Discussion

Delirium, characterized by acute cognitive and attentional dysfunction, poses a significant concern in healthcare. Its association with fluctuating consciousness levels and varied manifestations of agitation, lethargy, or mixed symptoms makes it particularly challenging. The aging population and increased life expectancy have led to a rise in postoperative delirium cases, including those following major surgeries or hip fractures [1,2]. Postoperative delirium adversely affects patient outcomes, leading to higher mortality rates [1], cognitive and functional decline [2], prolonged hospital stays, and increased healthcare costs. Despite clinical diagnosis using established manuals such as the DSM-V and ICD-10, there is a need for simpler and faster screening tools such as the CAM and delirium observation screening scales, albeit with lower sensitivity compared to expert assessments [3,4,5,6,7,8]. Various risk factors, including age, cognitive impairment, comorbidities, and medication use, contribute to postoperative delirium [9,10].

Effective management involves a multidisciplinary approach, encompassing preventive and therapeutic measures [11,12,13]. Non-pharmacological interventions emphasize creating a calm environment, patient orientation, early mobilization, optimizing physiological functions, and mindful breathing [14]. Intraoperative strategies involve lighter sedation and appropriate pain control, while postoperative care includes pain and medication reviews, infection monitoring, and metabolic assessment. The use of antipsychotics such as haloperidol and atypical agents such as risperidone has shown efficacy in treating delirium, though they carry side effects [21,22,23]. Risperidone, with its affinity for serotonin and dopamine receptors, offers advantages over traditional antipsychotics, making it a preferred first-line treatment [24,25]. Treatment decisions should prioritize using the minimum effective dose for the shortest necessary duration, particularly in severely agitated patients who threaten themselves or others.

Perioperative delirium is an important entity that affects senior patients. Although perioperative delirium was quite common in some studies, the incidence was relatively low in this study, with 3.7% of all patients older than 65 and 9.3% in the proximal femoral fracture group. Nevertheless, since perioperative delirium involves almost a tenth of the orthopedic surgery department’s patients and concerns the patient, family, and treating personnel, awareness and screening should be routine.

Delirium pathogenesis is still unknown, but a systemic review of the patient’s risk factors is warranted, recognizing past medical issues, electrolyte imbalance, drug treatment and sensual impairment. Although this practice makes sense, in this study we did not recognize physiological factors as affecting perioperative delirium development or duration. We found that the type of anesthesia influenced the timing and length of delirium. Regional anesthesia is related to earlier and shorter delirium, while general anesthesia is related to later and prolonged delirium. These findings suggest that perioperative delirium usually is not the consequence of a significant physiological impairment but instead due to an abrupt environmental change or the stress caused by the proximal femoral fracture and surgery performed. Further support for this theory is the lower delirium incidence in the elective surgery group, probably due to their being better prepared mentally for hospitalization. In this study, delirium tended to appear during the evening, 18:00 to 24:00, adding to the environmental aspect of delirium. 

We found that younger age correlates to a shorter delirium period, as expected, but did not influence delirium incidence. Higher blood albumin levels are also correlated to shorter delirium periods, maybe due to their role in buffering serum drug levels. 

There is no well-defined treatment protocol for postoperative delirium. Several drugs were used for treatment but with inconclusive results regarding efficiency. Our proposed rigid three-day low-dose risperidone treatment protocol, 0.5 mg BID, achieved 93.6% of delirium resolution within two days, as measured by CAM score normalization. A limitation of our study is the relatively small study group. We did not find that background diseases, medications and hemodialysis affect the development of postoperative delirium; the effect may be lower than expected and thus unnoticed in our study. Other limitations include the lack of delirium severity quantification and the lack of a control group. Although this is a relatively small observational and non-randomized study group, our low-dose risperidone treatment protocol suggests a clinical benefit and had no significant adverse effects. Earlier delirium resolution allows better physiotherapy adherence, earlier discharge and a better patient-family experience. 

## 5. Conclusions

Postoperative delirium in elderly patients suffering proximal femoral fractures is common. Treatment protocols are varied, inconclusive and conflicting. We recommend routine delirium screening, risk factor assessment and early management. We found in our observational study that the rigid three-day low-dose risperidone treatment protocol, 0.5 mg BID, was efficacious in delirium resolution, without side effects. Our treatment protocol showed promising results and should be further studied with a larger-scale RCT.

## Figures and Tables

**Table 1:** Patients’ characteristics.

Parameter	Number (%)
Study group	47
Males	22 (46.8)
Females	25 (53.2)
Age (years)	84.4 ± 8.6
BMI	25.4 ± 6.4
**Type of Surgery**	
Pertrochanteric Fracture Fixation	21 (44.7)
Femoral Arthroplasty	12 (25.5)
Subcapital Femoral Fracture Fixation	5 (10.6)
Conservative Treatment	3 (6.4)
Other Surgeries	6 (12.8)
Surgery Duration (mins)	56.3 ± 30.8
**Anaesthesia**	
General	27 (61.4)
Regional with Sedation	17 (38.6)
**Delirium**	
Delirium Onset from Hospitalization (days)	4 ± 6
Delirium Onset by Postoperative Day	2 ± 2
Initial CAM Score	3 ± 1
Days to Delirium Resolution	1.98 ± 0.68
CAM Score at the End of Treatment	0.11 ± 0.31

## Data Availability

The complete data are available under a confidentiality restriction.
